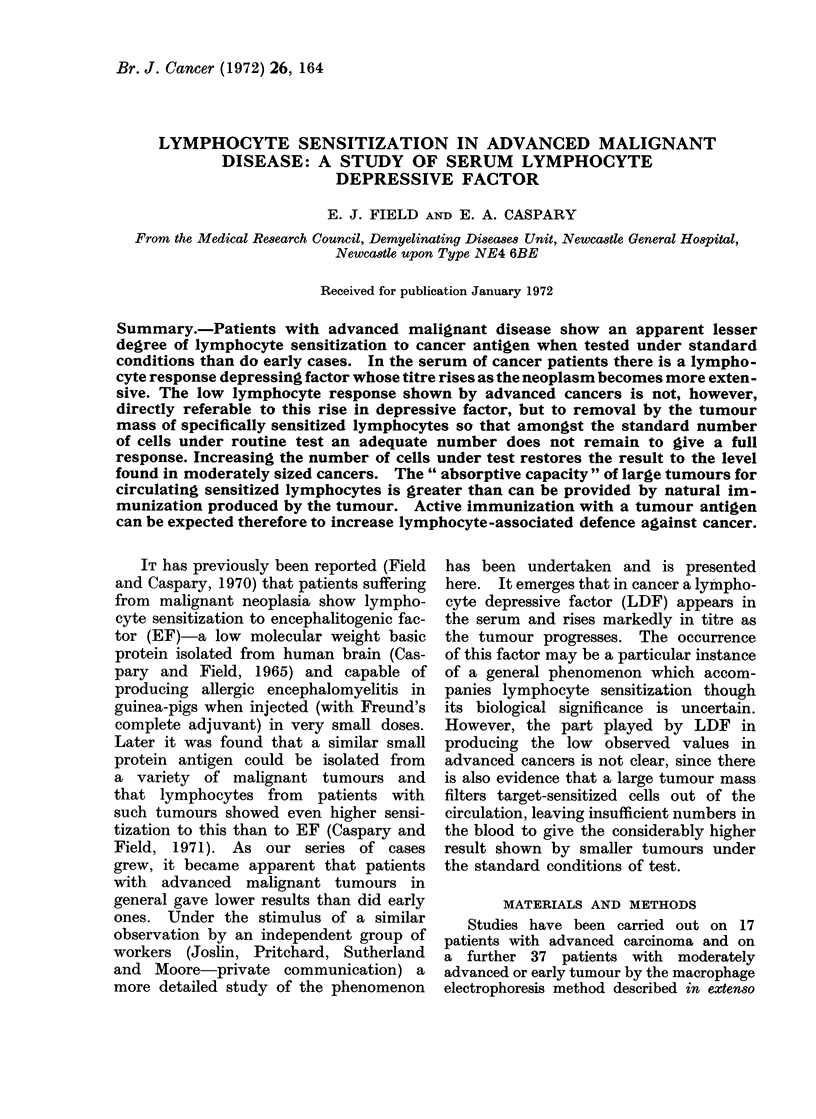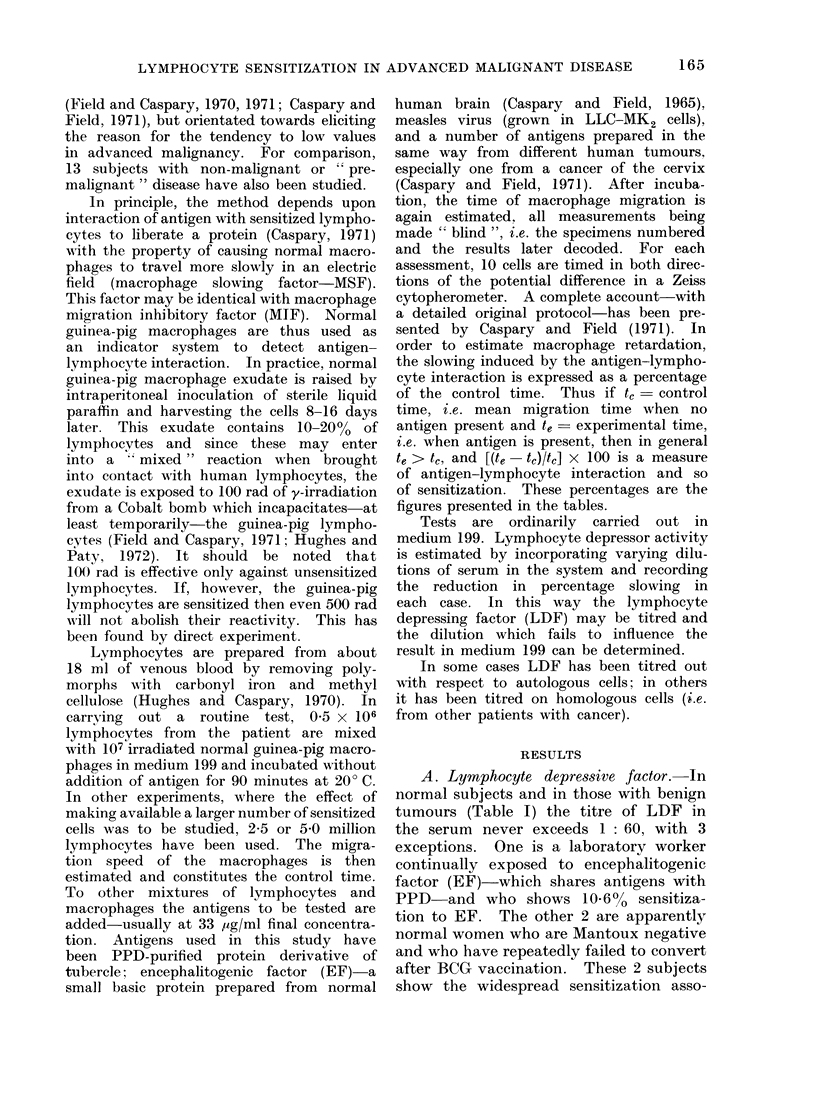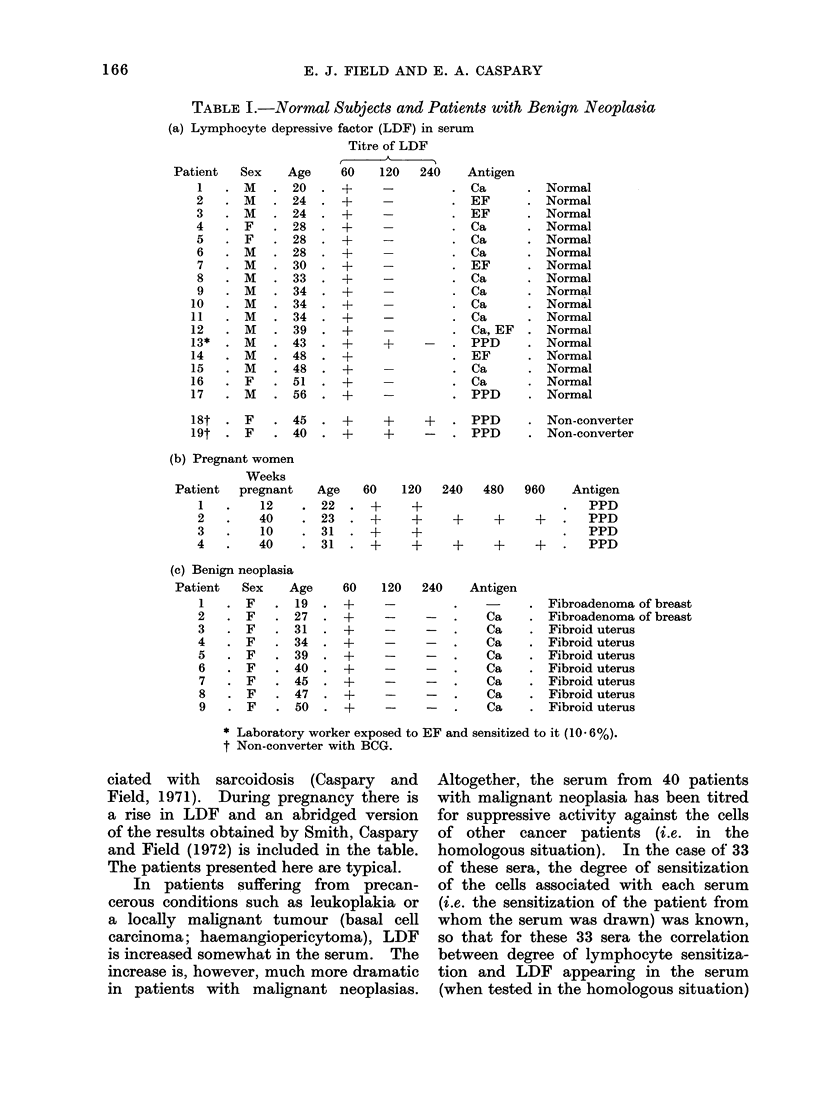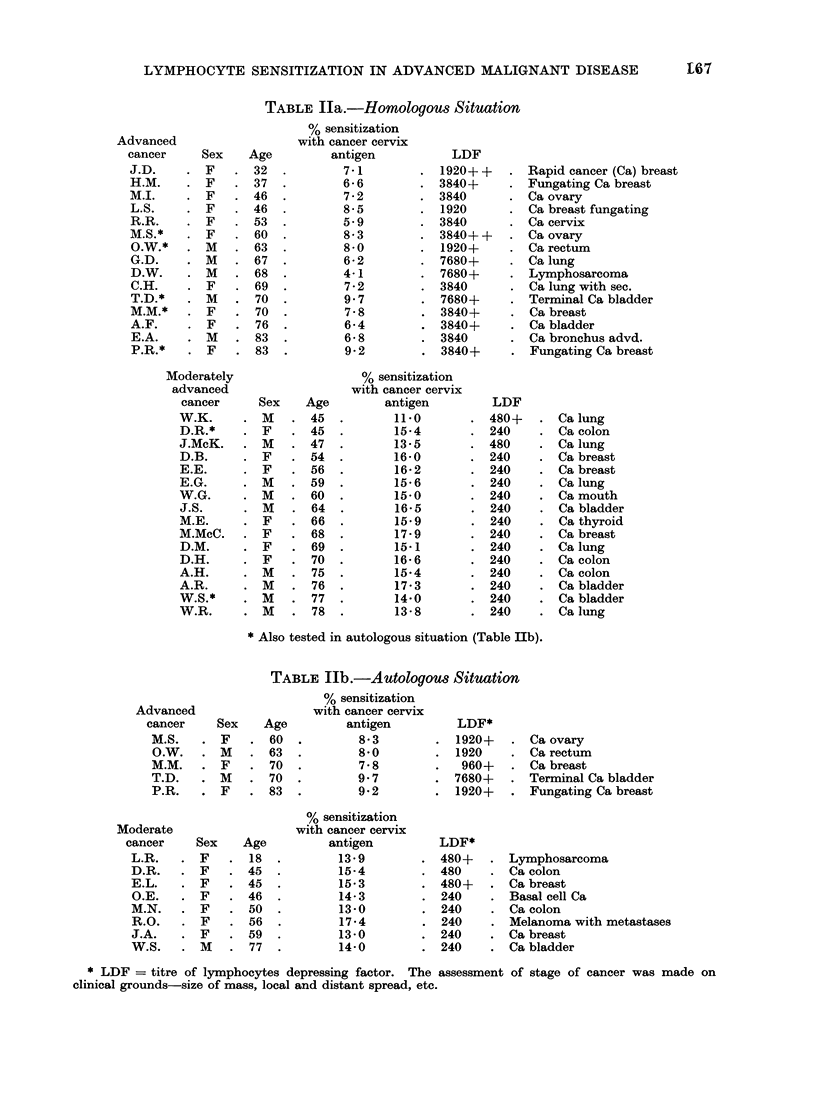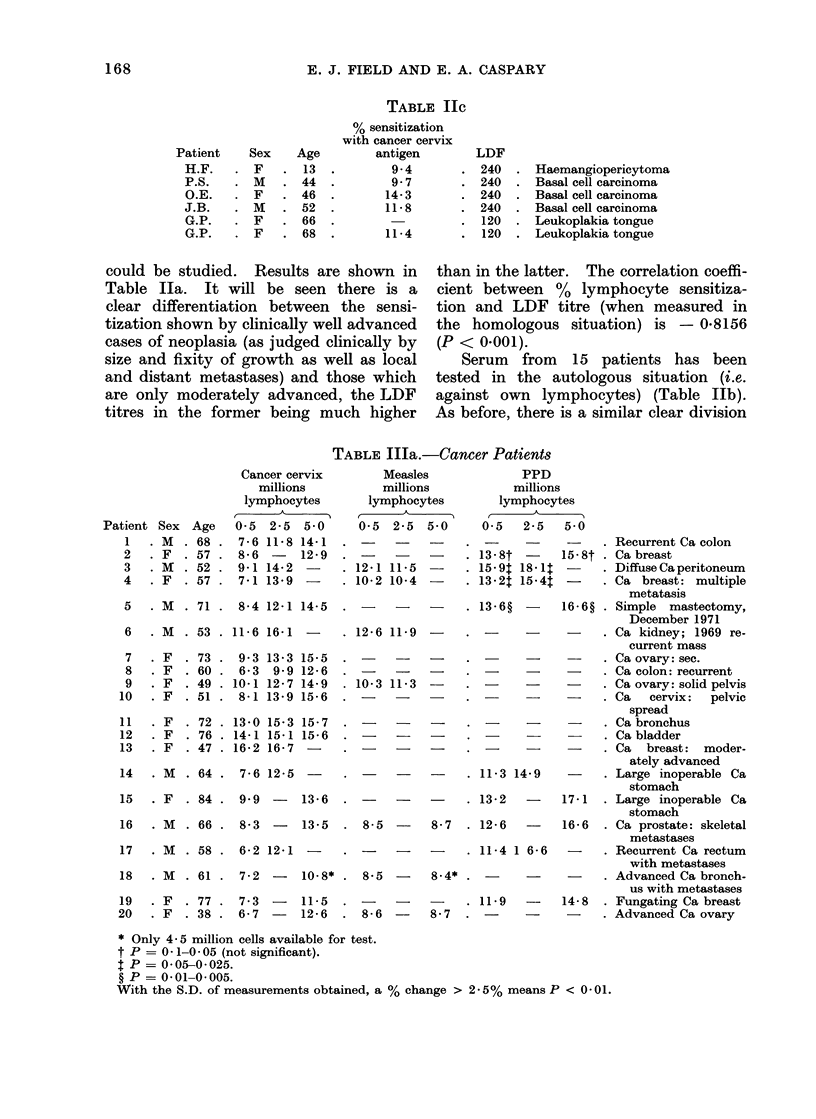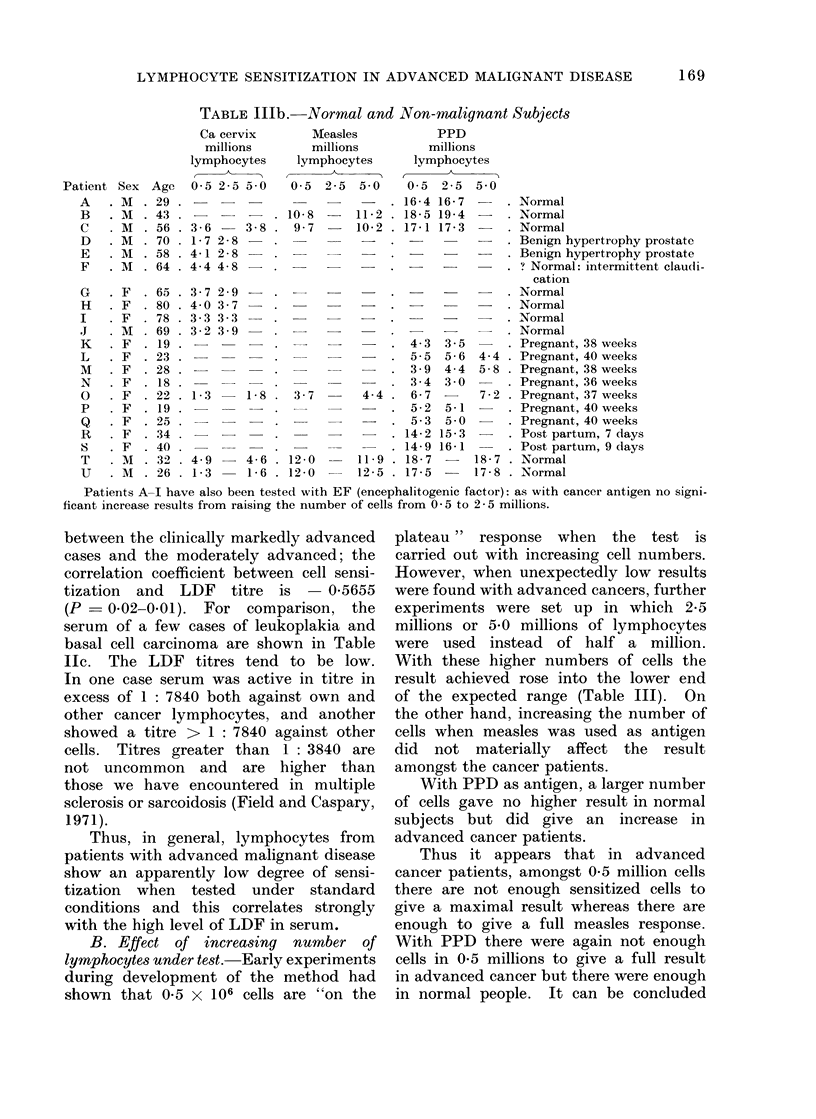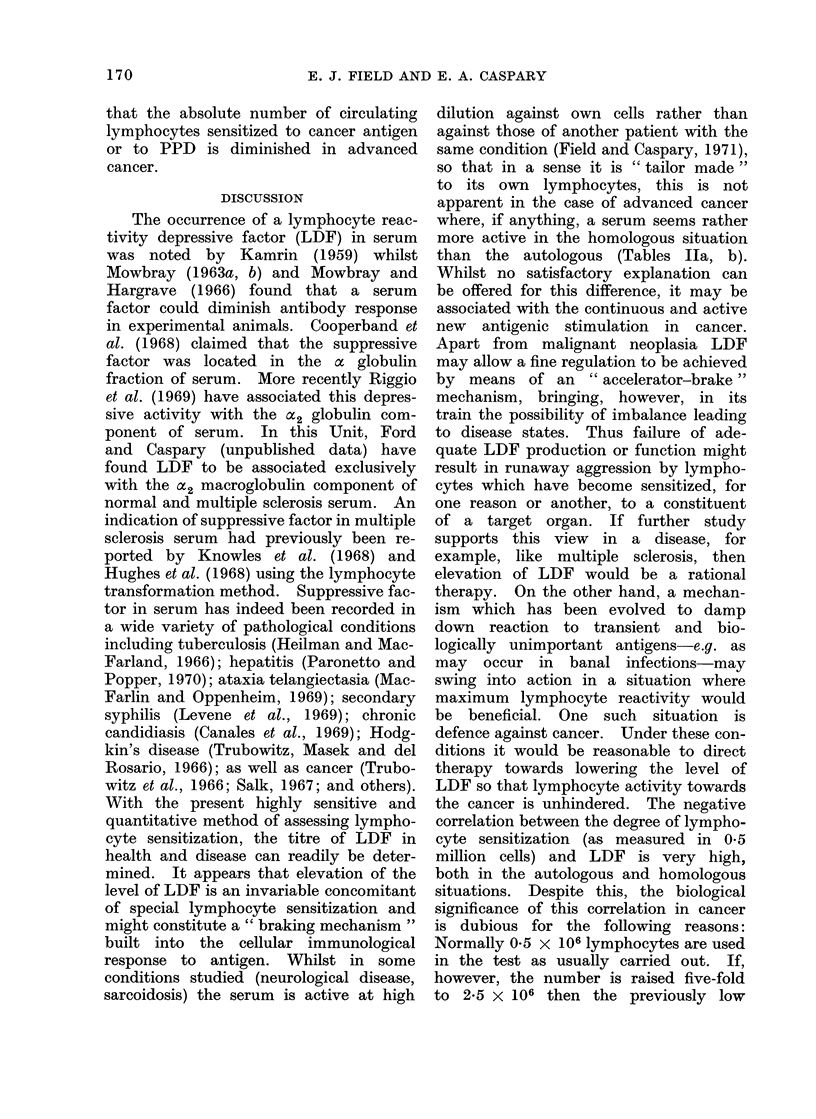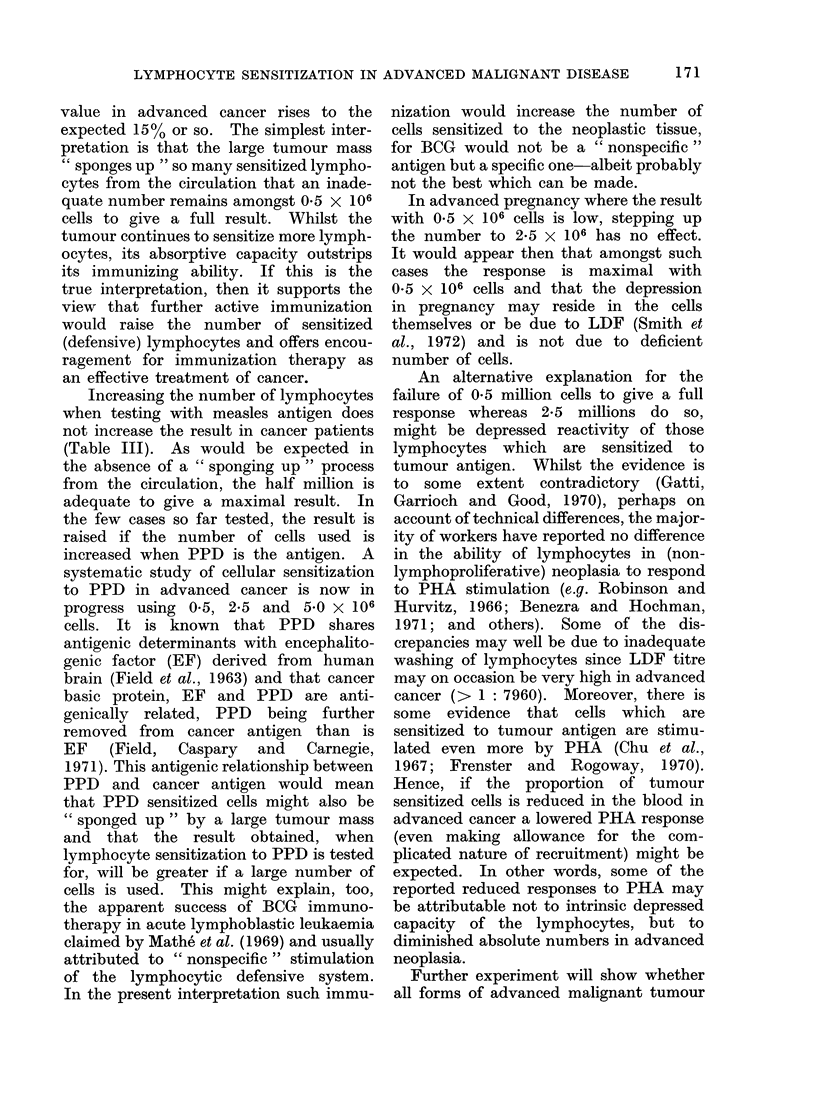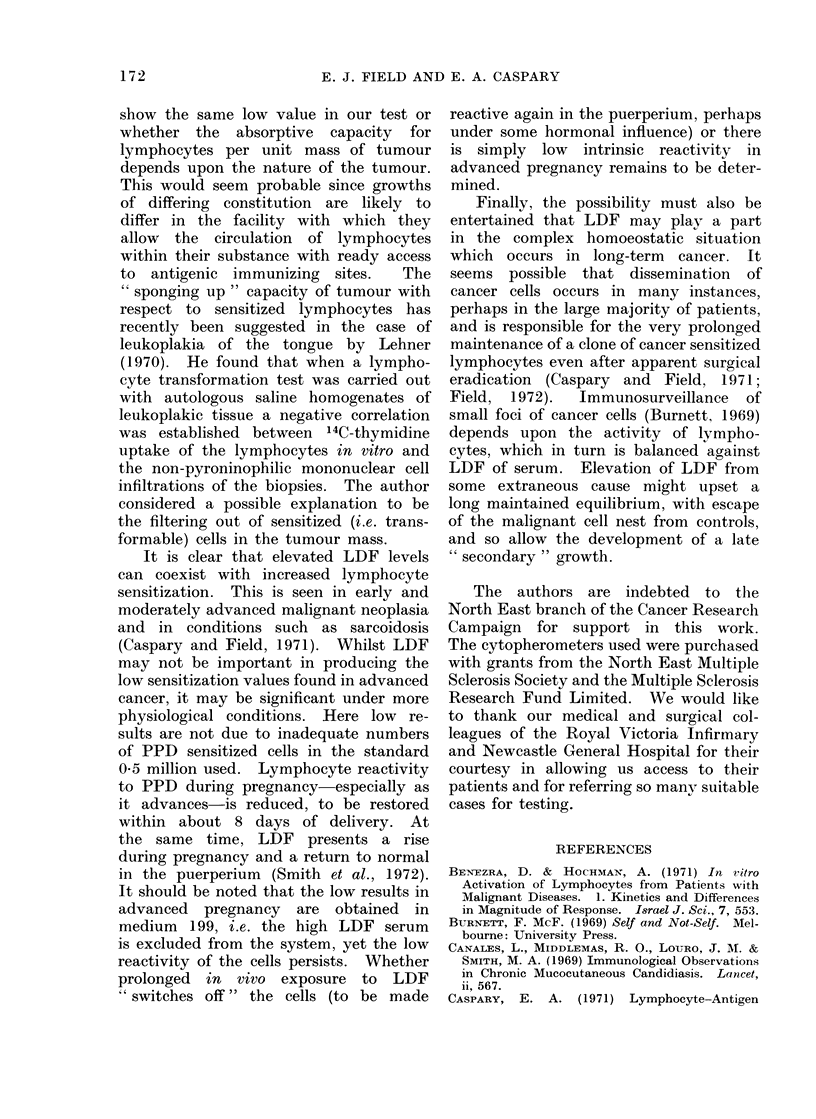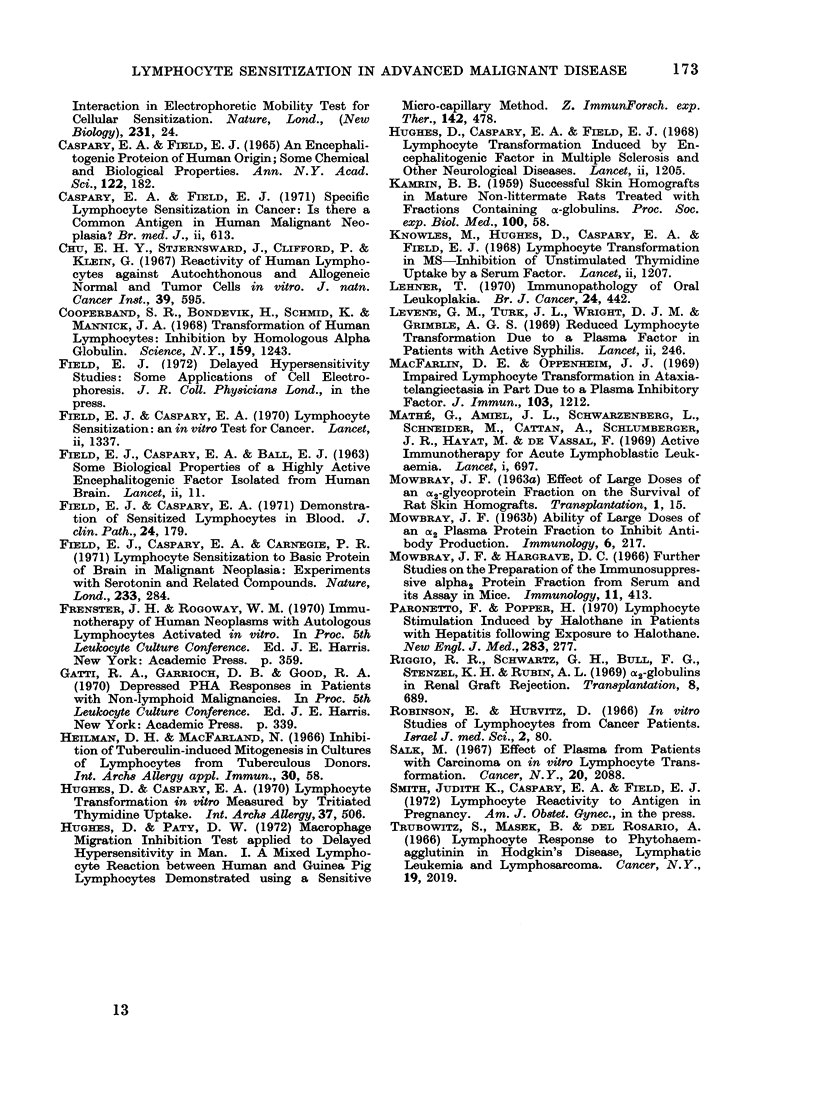# Lymphocyte Sensitization in Advanced Malignant Disease: A Study of Serum Lymphocyte Depressive Factor

**DOI:** 10.1038/bjc.1972.23

**Published:** 1972-06

**Authors:** E. J. Field, E. A. Caspary

## Abstract

Patients with advanced malignant disease show an apparent lesser degree of lymphocyte sensitization to cancer antigen when tested under standard conditions than do early cases. In the serum of cancer patients there is a lymphocyte response depressing factor whose titre rises as the neoplasm becomes more extensive. The low lymphocyte response shown by advanced cancers is not, however, directly referable to this rise in depressive factor, but to removal by the tumour mass of specifically sensitized lymphocytes so that amongst the standard number of cells under routine test an adequate number does not remain to give a full response. Increasing the number of cells under test restores the result to the level found in moderately sized cancers. The “absorptive capacity” of large tumours for circulating sensitized lymphocytes is greater than can be provided by natural immunization produced by the tumour. Active immunization with a tumour antigen can be expected therefore to increase lymphocyte-associated defence against cancer.


*Br. J. Cancer* (1972) **26**, 164

### LYMPHOCYTE SENSITIZATION IN ADVANCED MALIGNANT DISEASE: A STUDY OF SERUM LYMPHOCYTE DEPRESSIVE FACTOR

E. J. FIELD AND E. A. CASPARY

*From the Medical Research Council, Demyelinating Diseases Unit, Newcastle General Hospital, Newcastle upon Type NE4 6BE*

Received for publication January 1972

**Summary.—Patients with advanced malignant disease show an apparent lesser degree of lymphocyte sensitization to cancer antigen when tested under standard conditions than do early cases. In the serum of cancer patients there is a lymphocyte response depressing factor whose titre rises as the neoplasm becomes more extensive. The low lymphocyte response shown by advanced cancers is not, however, directly referable to this rise in depressive factor, but to removal by the tumour mass of specifically sensitized lymphocytes so that amongst the standard number of cells under routine test an adequate number does not remain to give a full response. Increasing the number of cells under test restores the result to the level found in moderately sized cancers. The " absorptive capacity " of large tumours for circulating sensitized lymphocytes is greater than can be provided by natural immunization produced by the tumour. Active immunization with a tumour antigen can be expected therefore to increase lymphocyte-associated defence against cancer.**

It has previously been reported (Field and Caspary, 1970) that patients suffering from malignant neoplasia show lymphocyte sensitization to encephalitogenic factor (EF)—a low molecular weight basic protein isolated from human brain (Caspary and Field, 1965) and capable of producing allergic encephalomyelitis in guinea-pigs when injected (with Freund's complete adjuvant) in very small doses. Later it was found that a similar small protein antigen could be isolated from a variety of malignant tumours and that lymphocytes from patients with such tumours showed even higher sensitization to this than to EF (Caspary and Field, 1971). As our series of cases grew, it became apparent that patients with advanced malignant tumours in general gave lower results than did early ones. Under the stimulus of a similar observation by an independent group of workers (Joslin, Pritchard, Sutherland and Moore—private communication) a more detailed study of the phenomenon has been undertaken and is presented here. It emerges that in cancer a lymphocyte depressive factor (LDF) appears in the serum and rises markedly in titre as the tumour progresses. The occurrence of this factor may be a particular instance of a general phenomenon which accompanies lymphocyte sensitization though its biological significance is uncertain. However, the part played by LDF in producing the low observed values in advanced cancers is not clear, since there is also evidence that a large tumour mass filters target-sensitized cells out of the circulation, leaving insufficient numbers in the blood to give the considerably higher result shown by smaller tumours under the standard conditions of test.

### MATERIALS AND METHODS

Studies have been carried out on 17 patients with advanced carcinoma and on a further 37 patients with moderately advanced or early tumour by the macrophage electrophoresis method described *in extenso*

(Field and Caspary, 1970, 1971; Caspary and Field, 1971), but orientated towards eliciting the reason for the tendency to low values in advanced malignancy. For comparison, 13 subjects with non-malignant or "pre-malignant" disease have also been studied.

In principle, the method depends upon interaction of antigen with sensitized lymphocytes to liberate a protein (Caspary, 1971) with the property of causing normal macrophages to travel more slowly in an electric field (macrophage slowing factor—MSF). This factor may be identical with macrophage migration inhibitory factor (MIF). Normal guinea-pig macrophages are thus used as an indicator system to detect antigen–lymphocyte interaction. In practice, normal guinea-pig macrophage exudate is raised by intraperitoneal inoculation of sterile liquid paraffin and harvesting the cells 8–16 days later. This exudate contains 10–20% of lymphocytes and since these may enter into a "mixed" reaction when brought into contact with human lymphocytes, the exudate is exposed to 100 rad of $\gamma$-irradiation from a Cobalt bomb which incapacitates—at least temporarily—the guinea-pig lymphocytes (Field and Caspary, 1971; Hughes and Paty, 1972). It should be noted that 100 rad is effective only against unsensitized lymphocytes. If, however, the guinea-pig lymphocytes are sensitized then even 500 rad will not abolish their reactivity. This has been found by direct experiment.

Lymphocytes are prepared from about 18 ml of venous blood by removing polymorphs with carbonyl iron and methyl cellulose (Hughes and Caspary, 1970). In carrying out a routine test, $0{\cdot}5 \times 10^6$ lymphocytes from the patient are mixed with $10^7$ irradiated normal guinea-pig macrophages in medium 199 and incubated without addition of antigen for 90 minutes at 20° C. In other experiments, where the effect of making available a larger number of sensitized cells was to be studied, 2·5 or 5·0 million lymphocytes have been used. The migration speed of the macrophages is then estimated and constitutes the control time. To other mixtures of lymphocytes and macrophages the antigens to be tested are added—usually at 33 $\mu$g/ml final concentration. Antigens used in this study have been PPD-purified protein derivative of tubercle; encephalitogenic factor (EF)—a small basic protein prepared from normal human brain (Caspary and Field, 1965), measles virus (grown in LLC-$MK_2$ cells), and a number of antigens prepared in the same way from different human tumours, especially one from a cancer of the cervix (Caspary and Field, 1971). After incubation, the time of macrophage migration is again estimated, all measurements being made "blind", *i.e.* the specimens numbered and the results later decoded. For each assessment, 10 cells are timed in both directions of the potential difference in a Zeiss cytopherometer. A complete account—with a detailed original protocol—has been presented by Caspary and Field (1971). In order to estimate macrophage retardation, the slowing induced by the antigen–lymphocyte interaction is expressed as a percentage of the control time. Thus if $t_c$ = control time, *i.e.* mean migration time when no antigen present and $t_e$ = experimental time, *i.e.* when antigen is present, then in general $t_e > t_c$, and $[(t_e - t_c)/t_c] \times 100$ is a measure of antigen–lymphocyte interaction and so of sensitization. These percentages are the figures presented in the tables.

Tests are ordinarily carried out in medium 199. Lymphocyte depressor activity is estimated by incorporating varying dilutions of serum in the system and recording the reduction in percentage slowing in each case. In this way the lymphocyte depressing factor (LDF) may be titred and the dilution which fails to influence the result in medium 199 can be determined.

In some cases LDF has been titred out with respect to autologous cells; in others it has been titred on homologous cells (*i.e.* from other patients with cancer).

### RESULTS

*A. Lymphocyte depressive factor.*—In normal subjects and in those with benign tumours (Table I) the titre of LDF in the serum never exceeds 1 : 60, with 3 exceptions. One is a laboratory worker continually exposed to encephalitogenic factor (EF)—which shares antigens with PPD—and who shows 10·6% sensitization to EF. The other 2 are apparently normal women who are Mantoux negative and who have repeatedly failed to convert after BCG vaccination. These 2 subjects show the widespread sensitization asso-

TABLE I.—*Normal Subjects and Patients with Benign Neoplasia*

(a) Lymphocyte depressive factor (LDF) in serum

| Patient | Sex | Age | Titre of LDF | | | Antigen | |
|---|---|---|---|---|---|---|---|
| | | | 60 | 120 | 240 | | |
| 1 | M | 20 | + | — | | Ca | Normal |
| 2 | M | 24 | + | — | | EF | Normal |
| 3 | M | 24 | + | — | | EF | Normal |
| 4 | F | 28 | + | — | | Ca | Normal |
| 5 | F | 28 | + | — | | Ca | Normal |
| 6 | M | 28 | + | — | | Ca | Normal |
| 7 | M | 30 | + | — | | EF | Normal |
| 8 | M | 33 | + | — | | Ca | Normal |
| 9 | M | 34 | + | — | | Ca | Normal |
| 10 | M | 34 | + | — | | Ca | Normal |
| 11 | M | 34 | + | — | | Ca | Normal |
| 12 | M | 39 | + | — | | Ca, EF | Normal |
| 13* | M | 43 | + | + | — | PPD | Normal |
| 14 | M | 48 | + | | | EF | Normal |
| 15 | M | 48 | + | — | | Ca | Normal |
| 16 | F | 51 | + | — | | Ca | Normal |
| 17 | M | 56 | + | — | | PPD | Normal |
| 18† | F | 45 | + | + | + | PPD | Non-converter |
| 19† | F | 40 | + | + | — | PPD | Non-converter |

(b) Pregnant women

| Patient | Weeks pregnant | Age | 60 | 120 | 240 | 480 | 960 | Antigen |
|---|---|---|---|---|---|---|---|---|
| 1 | 12 | 22 | + | + | | | | PPD |
| 2 | 40 | 23 | + | + | + | + | + | PPD |
| 3 | 10 | 31 | + | + | | | | PPD |
| 4 | 40 | 31 | + | + | + | + | + | PPD |

(c) Benign neoplasia

| Patient | Sex | Age | 60 | 120 | 240 | Antigen | |
|---|---|---|---|---|---|---|---|
| 1 | F | 19 | + | — | | — | Fibroadenoma of breast |
| 2 | F | 27 | + | — | — | Ca | Fibroadenoma of breast |
| 3 | F | 31 | + | — | — | Ca | Fibroid uterus |
| 4 | F | 34 | + | — | — | Ca | Fibroid uterus |
| 5 | F | 39 | + | — | — | Ca | Fibroid uterus |
| 6 | F | 40 | + | — | — | Ca | Fibroid uterus |
| 7 | F | 45 | + | — | — | Ca | Fibroid uterus |
| 8 | F | 47 | + | — | — | Ca | Fibroid uterus |
| 9 | F | 50 | + | — | — | Ca | Fibroid uterus |

* Laboratory worker exposed to EF and sensitized to it (10·6%).
† Non-converter with BCG.

ciated with sarcoidosis (Caspary and Field, 1971). During pregnancy there is a rise in LDF and an abridged version of the results obtained by Smith, Caspary and Field (1972) is included in the table. The patients presented here are typical.

In patients suffering from precancerous conditions such as leukoplakia or a locally malignant tumour (basal cell carcinoma; haemangiopericytoma), LDF is increased somewhat in the serum. The increase is, however, much more dramatic in patients with malignant neoplasias. Altogether, the serum from 40 patients with malignant neoplasia has been titred for suppressive activity against the cells of other cancer patients (*i.e.* in the homologous situation). In the case of 33 of these sera, the degree of sensitization of the cells associated with each serum (*i.e.* the sensitization of the patient from whom the serum was drawn) was known, so that for these 33 sera the correlation between degree of lymphocyte sensitization and LDF appearing in the serum (when tested in the homologous situation)

TABLE IIa.—*Homologous Situation*

| Advanced cancer | Sex | Age | % sensitization with cancer cervix antigen | LDF | |
|---|---|---|---|---|---|
| J.D. | F | 32 | 7·1 | 1920++ | Rapid cancer (Ca) breast |
| H.M. | F | 37 | 6·6 | 3840+ | Fungating Ca breast |
| M.I. | F | 46 | 7·2 | 3840 | Ca ovary |
| L.S. | F | 46 | 8·5 | 1920 | Ca breast fungating |
| R.R. | F | 53 | 5·9 | 3840 | Ca cervix |
| M.S.* | F | 60 | 8·3 | 3840++ | Ca ovary |
| O.W.* | M | 63 | 8·0 | 1920+ | Ca rectum |
| G.D. | M | 67 | 6·2 | 7680+ | Ca lung |
| D.W. | M | 68 | 4·1 | 7680+ | Lymphosarcoma |
| C.H. | F | 69 | 7·2 | 3840 | Ca lung with sec. |
| T.D.* | M | 70 | 9·7 | 7680+ | Terminal Ca bladder |
| M.M.* | F | 70 | 7·8 | 3840+ | Ca breast |
| A.F. | F | 76 | 6·4 | 3840+ | Ca bladder |
| E.A. | M | 83 | 6·8 | 3840 | Ca bronchus advd. |
| P.R.* | F | 83 | 9·2 | 3840+ | Fungating Ca breast |

| Moderately advanced cancer | Sex | Age | % sensitization with cancer cervix antigen | LDF | |
|---|---|---|---|---|---|
| W.K. | M | 45 | 11·0 | 480+ | Ca lung |
| D.R.* | F | 45 | 15·4 | 240 | Ca colon |
| J.McK. | M | 47 | 13·5 | 480 | Ca lung |
| D.B. | F | 54 | 16·0 | 240 | Ca breast |
| E.E. | F | 56 | 16·2 | 240 | Ca breast |
| E.G. | M | 59 | 15·6 | 240 | Ca lung |
| W.G. | M | 60 | 15·0 | 240 | Ca mouth |
| J.S. | M | 64 | 16·5 | 240 | Ca bladder |
| M.E. | F | 66 | 15·9 | 240 | Ca thyroid |
| M.McC. | F | 68 | 17·9 | 240 | Ca breast |
| D.M. | F | 69 | 15·1 | 240 | Ca lung |
| D.H. | F | 70 | 16·6 | 240 | Ca colon |
| A.H. | M | 75 | 15·4 | 240 | Ca colon |
| A.R. | M | 76 | 17·3 | 240 | Ca bladder |
| W.S.* | M | 77 | 14·0 | 240 | Ca bladder |
| W.R. | M | 78 | 13·8 | 240 | Ca lung |

* Also tested in autologous situation (Table IIb).

TABLE IIb.—*Autologous Situation*

| Advanced cancer | Sex | Age | % sensitization with cancer cervix antigen | LDF* | |
|---|---|---|---|---|---|
| M.S. | F | 60 | 8·3 | 1920+ | Ca ovary |
| O.W. | M | 63 | 8·0 | 1920 | Ca rectum |
| M.M. | F | 70 | 7·8 | 960+ | Ca breast |
| T.D. | M | 70 | 9·7 | 7680+ | Terminal Ca bladder |
| P.R. | F | 83 | 9·2 | 1920+ | Fungating Ca breast |

| Moderate cancer | Sex | Age | % sensitization with cancer cervix antigen | LDF* | |
|---|---|---|---|---|---|
| L.R. | F | 18 | 13·9 | 480+ | Lymphosarcoma |
| D.R. | F | 45 | 15·4 | 480 | Ca colon |
| E.L. | F | 45 | 15·3 | 480+ | Ca breast |
| O.E. | F | 46 | 14·3 | 240 | Basal cell Ca |
| M.N. | F | 50 | 13·0 | 240 | Ca colon |
| R.O. | F | 56 | 17·4 | 240 | Melanoma with metastases |
| J.A. | F | 59 | 13·0 | 240 | Ca breast |
| W.S. | M | 77 | 14·0 | 240 | Ca bladder |

* LDF = titre of lymphocytes depressing factor. The assessment of stage of cancer was made on clinical grounds—size of mass, local and distant spread, etc.

TABLE IIc

| Patient | Sex | Age | % sensitization with cancer cervix antigen | LDF | |
|---|---|---|---|---|---|
| H.F. | F | 13 | 9·4 | 240 | Haemangiopericytoma |
| P.S. | M | 44 | 9·7 | 240 | Basal cell carcinoma |
| O.E. | F | 46 | 14·3 | 240 | Basal cell carcinoma |
| J.B. | M | 52 | 11·8 | 240 | Basal cell carcinoma |
| G.P. | F | 66 | — | 120 | Leukoplakia tongue |
| G.P. | F | 68 | 11·4 | 120 | Leukoplakia tongue |

could be studied. Results are shown in Table IIa. It will be seen there is a clear differentiation between the sensitization shown by clinically well advanced cases of neoplasia (as judged clinically by size and fixity of growth as well as local and distant metastases) and those which are only moderately advanced, the LDF titres in the former being much higher than in the latter. The correlation coefficient between % lymphocyte sensitization and LDF titre (when measured in the homologous situation) is — 0·8156 ($P < 0{\cdot}001$).

Serum from 15 patients has been tested in the autologous situation (*i.e.* against own lymphocytes) (Table IIb). As before, there is a similar clear division

TABLE IIIa.—*Cancer Patients*

| | | | Cancer cervix millions lymphocytes | | | Measles millions lymphocytes | | | PPD millions lymphocytes | | | |
|---|---|---|---|---|---|---|---|---|---|---|---|---|
| Patient | Sex | Age | 0·5 | 2·5 | 5·0 | 0·5 | 2·5 | 5·0 | 0·5 | 2·5 | 5·0 | |
| 1 | M | 68 | 7·6 | 11·8 | 14·1 | — | — | — | — | — | — | Recurrent Ca colon |
| 2 | F | 57 | 8·6 | — | 12·9 | — | — | — | 13·8† | — | 15·8† | Ca breast |
| 3 | M | 52 | 9·1 | 14·2 | — | 12·1 | 11·5 | — | 15·9‡ | 18·1‡ | — | Diffuse Ca peritoneum |
| 4 | F | 57 | 7·1 | 13·9 | — | 10·2 | 10·4 | — | 13·2‡ | 15·4‡ | — | Ca breast: multiple metatasis |
| 5 | M | 71 | 8·4 | 12·1 | 14·5 | — | — | — | 13·6§ | — | 16·6§ | Simple mastectomy, December 1971 |
| 6 | M | 53 | 11·6 | 16·1 | — | 12·6 | 11·9 | — | — | — | — | Ca kidney; 1969 recurrent mass |
| 7 | F | 73 | 9·3 | 13·3 | 15·5 | — | — | — | — | — | — | Ca ovary: sec. |
| 8 | F | 60 | 6·3 | 9·9 | 12·6 | — | — | — | — | — | — | Ca colon: recurrent |
| 9 | F | 49 | 10·1 | 12·7 | 14·9 | 10·3 | 11·3 | — | — | — | — | Ca ovary: solid pelvis |
| 10 | F | 51 | 8·1 | 13·9 | 15·6 | — | — | — | — | — | — | Ca cervix: pelvic spread |
| 11 | F | 72 | 13·0 | 15·3 | 15·7 | — | — | — | — | — | — | Ca bronchus |
| 12 | F | 76 | 14·1 | 15·1 | 15·6 | — | — | — | — | — | — | Ca bladder |
| 13 | F | 47 | 16·2 | 16·7 | — | — | — | — | — | — | — | Ca breast: moderately advanced |
| 14 | M | 64 | 7·6 | 12·5 | — | — | — | — | 11·3 | 14·9 | — | Large inoperable Ca stomach |
| 15 | F | 84 | 9·9 | — | 13·6 | — | — | — | 13·2 | — | 17·1 | Large inoperable Ca stomach |
| 16 | M | 66 | 8·3 | — | 13·5 | 8·5 | — | 8·7 | 12·6 | — | 16·6 | Ca prostate: skeletal metastases |
| 17 | M | 58 | 6·2 | 12·1 | — | — | — | — | 11·4 | 1 6·6 | — | Recurrent Ca rectum with metastases |
| 18 | M | 61 | 7·2 | — | 10·8* | 8·5 | — | 8·4* | — | — | — | Advanced Ca bronchus with metastases |
| 19 | F | 77 | 7·3 | — | 11·5 | — | — | — | 11·9 | — | 14·8 | Fungating Ca breast |
| 20 | F | 38 | 6·7 | — | 12·6 | 8·6 | — | 8·7 | — | — | — | Advanced Ca ovary |

* Only 4·5 million cells available for test.
† $P = 0{\cdot}1$–$0{\cdot}05$ (not significant).
‡ $P = 0{\cdot}05$–$0{\cdot}025$.
§ $P = 0{\cdot}01$–$0{\cdot}005$.
With the S.D. of measurements obtained, a % change > 2·5% means $P < 0{\cdot}01$.

TABLE IIIb.—*Normal and Non-malignant Subjects*

| Patient | Sex | Age | Ca cervix millions lymphocytes 0·5 | 2·5 | 5·0 | Measles millions lymphocytes 0·5 | 2·5 | 5·0 | PPD millions lymphocytes 0·5 | 2·5 | 5·0 | |
|---|---|---|---|---|---|---|---|---|---|---|---|---|
| A | M | 29 | — | — | — | — | — | — | 16·4 | 16·7 | — | Normal |
| B | M | 43 | — | — | — | 10·8 | — | 11·2 | 18·5 | 19·4 | — | Normal |
| C | M | 56 | 3·6 | — | 3·8 | 9·7 | — | 10·2 | 17·1 | 17·3 | — | Normal |
| D | M | 70 | 1·7 | 2·8 | — | — | — | — | — | — | — | Benign hypertrophy prostate |
| E | M | 58 | 4·1 | 2·8 | — | — | — | — | — | — | — | Benign hypertrophy prostate |
| F | M | 64 | 4·4 | 4·8 | — | — | — | — | — | — | — | ? Normal: intermittent claudication |
| G | F | 65 | 3·7 | 2·9 | — | — | — | — | — | — | — | Normal |
| H | F | 80 | 4·0 | 3·7 | — | — | — | — | — | — | — | Normal |
| I | F | 78 | 3·3 | 3·3 | — | — | — | — | — | — | — | Normal |
| J | M | 69 | 3·2 | 3·9 | — | — | — | — | — | — | — | Normal |
| K | F | 19 | — | — | — | — | — | — | 4·3 | 3·5 | — | Pregnant, 38 weeks |
| L | F | 23 | — | — | — | — | — | — | 5·5 | 5·6 | 4·4 | Pregnant, 40 weeks |
| M | F | 28 | — | — | — | — | — | — | 3·9 | 4·4 | 5·8 | Pregnant, 38 weeks |
| N | F | 18 | — | — | — | — | — | — | 3·4 | 3·0 | — | Pregnant, 36 weeks |
| O | F | 22 | 1·3 | — | 1·8 | 3·7 | — | 4·4 | 6·7 | — | 7·2 | Pregnant, 37 weeks |
| P | F | 19 | — | — | — | — | — | — | 5·2 | 5·1 | — | Pregnant, 40 weeks |
| Q | F | 25 | — | — | — | — | — | — | 5·3 | 5·0 | — | Pregnant, 40 weeks |
| R | F | 34 | — | — | — | — | — | — | 14·2 | 15·3 | — | Post partum, 7 days |
| S | F | 40 | — | — | — | — | — | — | 14·9 | 16·1 | — | Post partum, 9 days |
| T | M | 32 | 4·9 | — | 4·6 | 12·0 | — | 11·9 | 18·7 | — | 18·7 | Normal |
| U | M | 26 | 1·3 | — | 1·6 | 12·0 | — | 12·5 | 17·5 | — | 17·8 | Normal |

Patients A–I have also been tested with EF (encephalitogenic factor): as with cancer antigen no significant increase results from raising the number of cells from 0·5 to 2·5 millions.

between the clinically markedly advanced cases and the moderately advanced; the correlation coefficient between cell sensitization and LDF titre is — 0·5655 ($P$ = 0·02–0·01). For comparison, the serum of a few cases of leukoplakia and basal cell carcinoma are shown in Table IIc. The LDF titres tend to be low. In one case serum was active in titre in excess of 1 : 7840 both against own and other cancer lymphocytes, and another showed a titre > 1 : 7840 against other cells. Titres greater than 1 : 3840 are not uncommon and are higher than those we have encountered in multiple sclerosis or sarcoidosis (Field and Caspary, 1971).

Thus, in general, lymphocytes from patients with advanced malignant disease show an apparently low degree of sensitization when tested under standard conditions and this correlates strongly with the high level of LDF in serum.

*B. Effect of increasing number of lymphocytes under test.*—Early experiments during development of the method had shown that $0·5 \times 10^6$ cells are "on the plateau" response when the test is carried out with increasing cell numbers. However, when unexpectedly low results were found with advanced cancers, further experiments were set up in which 2·5 millions or 5·0 millions of lymphocytes were used instead of half a million. With these higher numbers of cells the result achieved rose into the lower end of the expected range (Table III). On the other hand, increasing the number of cells when measles was used as antigen did not materially affect the result amongst the cancer patients.

With PPD as antigen, a larger number of cells gave no higher result in normal subjects but did give an increase in advanced cancer patients.

Thus it appears that in advanced cancer patients, amongst 0·5 million cells there are not enough sensitized cells to give a maximal result whereas there are enough to give a full measles response. With PPD there were again not enough cells in 0·5 millions to give a full result in advanced cancer but there were enough in normal people. It can be concluded that the absolute number of circulating lymphocytes sensitized to cancer antigen or to PPD is diminished in advanced cancer.

### DISCUSSION

The occurrence of a lymphocyte reactivity depressive factor (LDF) in serum was noted by Kamrin (1959) whilst Mowbray (1963*a*, *b*) and Mowbray and Hargrave (1966) found that a serum factor could diminish antibody response in experimental animals. Cooperband *et al.* (1968) claimed that the suppressive factor was located in the $\alpha$ globulin fraction of serum. More recently Riggio *et al.* (1969) have associated this depressive activity with the $\alpha_2$ globulin component of serum. In this Unit, Ford and Caspary (unpublished data) have found LDF to be associated exclusively with the $\alpha_2$ macroglobulin component of normal and multiple sclerosis serum. An indication of suppressive factor in multiple sclerosis serum had previously been reported by Knowles *et al.* (1968) and Hughes *et al.* (1968) using the lymphocyte transformation method. Suppressive factor in serum has indeed been recorded in a wide variety of pathological conditions including tuberculosis (Heilman and MacFarland, 1966); hepatitis (Paronetto and Popper, 1970); ataxia telangiectasia (MacFarlin and Oppenheim, 1969); secondary syphilis (Levene *et al.*, 1969); chronic candidiasis (Canales *et al.*, 1969); Hodgkin's disease (Trubowitz, Masek and del Rosario, 1966); as well as cancer (Trubowitz *et al.*, 1966; Salk, 1967; and others). With the present highly sensitive and quantitative method of assessing lymphocyte sensitization, the titre of LDF in health and disease can readily be determined. It appears that elevation of the level of LDF is an invariable concomitant of special lymphocyte sensitization and might constitute a " braking mechanism " built into the cellular immunological response to antigen. Whilst in some conditions studied (neurological disease, sarcoidosis) the serum is active at high dilution against own cells rather than against those of another patient with the same condition (Field and Caspary, 1971), so that in a sense it is " tailor made " to its own lymphocytes, this is not apparent in the case of advanced cancer where, if anything, a serum seems rather more active in the homologous situation than the autologous (Tables IIa, b). Whilst no satisfactory explanation can be offered for this difference, it may be associated with the continuous and active new antigenic stimulation in cancer. Apart from malignant neoplasia LDF may allow a fine regulation to be achieved by means of an " accelerator–brake " mechanism, bringing, however, in its train the possibility of imbalance leading to disease states. Thus failure of adequate LDF production or function might result in runaway aggression by lymphocytes which have become sensitized, for one reason or another, to a constituent of a target organ. If further study supports this view in a disease, for example, like multiple sclerosis, then elevation of LDF would be a rational therapy. On the other hand, a mechanism which has been evolved to damp down reaction to transient and biologically unimportant antigens—*e.g.* as may occur in banal infections—may swing into action in a situation where maximum lymphocyte reactivity would be beneficial. One such situation is defence against cancer. Under these conditions it would be reasonable to direct therapy towards lowering the level of LDF so that lymphocyte activity towards the cancer is unhindered. The negative correlation between the degree of lymphocyte sensitization (as measured in 0·5 million cells) and LDF is very high, both in the autologous and homologous situations. Despite this, the biological significance of this correlation in cancer is dubious for the following reasons: Normally $0{\cdot}5 \times 10^6$ lymphocytes are used in the test as usually carried out. If, however, the number is raised five-fold to $2{\cdot}5 \times 10^6$ then the previously low value in advanced cancer rises to the expected 15% or so. The simplest interpretation is that the large tumour mass "sponges up" so many sensitized lymphocytes from the circulation that an inadequate number remains amongst $0{\cdot}5 \times 10^6$ cells to give a full result. Whilst the tumour continues to sensitize more lymphocytes, its absorptive capacity outstrips its immunizing ability. If this is the true interpretation, then it supports the view that further active immunization would raise the number of sensitized (defensive) lymphocytes and offers encouragement for immunization therapy as an effective treatment of cancer.

Increasing the number of lymphocytes when testing with measles antigen does not increase the result in cancer patients (Table III). As would be expected in the absence of a "sponging up" process from the circulation, the half million is adequate to give a maximal result. In the few cases so far tested, the result is raised if the number of cells used is increased when PPD is the antigen. A systematic study of cellular sensitization to PPD in advanced cancer is now in progress using 0·5, 2·5 and $5{\cdot}0 \times 10^6$ cells. It is known that PPD shares antigenic determinants with encephalitogenic factor (EF) derived from human brain (Field *et al.*, 1963) and that cancer basic protein, EF and PPD are antigenically related, PPD being further removed from cancer antigen than is EF (Field, Caspary and Carnegie, 1971). This antigenic relationship between PPD and cancer antigen would mean that PPD sensitized cells might also be "sponged up" by a large tumour mass and that the result obtained, when lymphocyte sensitization to PPD is tested for, will be greater if a large number of cells is used. This might explain, too, the apparent success of BCG immunotherapy in acute lymphoblastic leukaemia claimed by Mathé *et al.* (1969) and usually attributed to "nonspecific" stimulation of the lymphocytic defensive system. In the present interpretation such immunization would increase the number of cells sensitized to the neoplastic tissue, for BCG would not be a "nonspecific" antigen but a specific one—albeit probably not the best which can be made.

In advanced pregnancy where the result with $0{\cdot}5 \times 10^6$ cells is low, stepping up the number to $2{\cdot}5 \times 10^6$ has no effect. It would appear then that amongst such cases the response is maximal with $0{\cdot}5 \times 10^6$ cells and that the depression in pregnancy may reside in the cells themselves or be due to LDF (Smith *et al.*, 1972) and is not due to deficient number of cells.

An alternative explanation for the failure of 0·5 million cells to give a full response whereas 2·5 millions do so, might be depressed reactivity of those lymphocytes which are sensitized to tumour antigen. Whilst the evidence is to some extent contradictory (Gatti, Garrioch and Good, 1970), perhaps on account of technical differences, the majority of workers have reported no difference in the ability of lymphocytes in (nonlymphoproliferative) neoplasia to respond to PHA stimulation (*e.g.* Robinson and Hurvitz, 1966; Benezra and Hochman, 1971; and others). Some of the discrepancies may well be due to inadequate washing of lymphocytes since LDF titre may on occasion be very high in advanced cancer ($> 1 : 7960$). Moreover, there is some evidence that cells which are sensitized to tumour antigen are stimulated even more by PHA (Chu *et al.*, 1967; Frenster and Rogoway, 1970). Hence, if the proportion of tumour sensitized cells is reduced in the blood in advanced cancer a lowered PHA response (even making allowance for the complicated nature of recruitment) might be expected. In other words, some of the reported reduced responses to PHA may be attributable not to intrinsic depressed capacity of the lymphocytes, but to diminished absolute numbers in advanced neoplasia.

Further experiment will show whether all forms of advanced malignant tumour show the same low value in our test or whether the absorptive capacity for lymphocytes per unit mass of tumour depends upon the nature of the tumour. This would seem probable since growths of differing constitution are likely to differ in the facility with which they allow the circulation of lymphocytes within their substance with ready access to antigenic immunizing sites. The " sponging up " capacity of tumour with respect to sensitized lymphocytes has recently been suggested in the case of leukoplakia of the tongue by Lehner (1970). He found that when a lymphocyte transformation test was carried out with autologous saline homogenates of leukoplakic tissue a negative correlation was established between $^{14}C$-thymidine uptake of the lymphocytes *in vitro* and the non-pyroninophilic mononuclear cell infiltrations of the biopsies. The author considered a possible explanation to be the filtering out of sensitized (*i.e.* transformable) cells in the tumour mass.

It is clear that elevated LDF levels can coexist with increased lymphocyte sensitization. This is seen in early and moderately advanced malignant neoplasia and in conditions such as sarcoidosis (Caspary and Field, 1971). Whilst LDF may not be important in producing the low sensitization values found in advanced cancer, it may be significant under more physiological conditions. Here low results are not due to inadequate numbers of PPD sensitized cells in the standard 0·5 million used. Lymphocyte reactivity to PPD during pregnancy—especially as it advances—is reduced, to be restored within about 8 days of delivery. At the same time, LDF presents a rise during pregnancy and a return to normal in the puerperium (Smith *et al.*, 1972). It should be noted that the low results in advanced pregnancy are obtained in medium 199, *i.e.* the high LDF serum is excluded from the system, yet the low reactivity of the cells persists. Whether prolonged *in vivo* exposure to LDF " switches off " the cells (to be made reactive again in the puerperium, perhaps under some hormonal influence) or there is simply low intrinsic reactivity in advanced pregnancy remains to be determined.

Finally, the possibility must also be entertained that LDF may play a part in the complex homoeostatic situation which occurs in long-term cancer. It seems possible that dissemination of cancer cells occurs in many instances, perhaps in the large majority of patients, and is responsible for the very prolonged maintenance of a clone of cancer sensitized lymphocytes even after apparent surgical eradication (Caspary and Field, 1971; Field, 1972). Immunosurveillance of small foci of cancer cells (Burnett, 1969) depends upon the activity of lymphocytes, which in turn is balanced against LDF of serum. Elevation of LDF from some extraneous cause might upset a long maintained equilibrium, with escape of the malignant cell nest from controls, and so allow the development of a late " secondary " growth.

The authors are indebted to the North East branch of the Cancer Research Campaign for support in this work. The cytopherometers used were purchased with grants from the North East Multiple Sclerosis Society and the Multiple Sclerosis Research Fund Limited. We would like to thank our medical and surgical colleagues of the Royal Victoria Infirmary and Newcastle General Hospital for their courtesy in allowing us access to their patients and for referring so many suitable cases for testing.

### REFERENCES

Benezra, D. & Hochman, A. (1971) *In vitro* Activation of Lymphocytes from Patients with Malignant Diseases. 1. Kinetics and Differences in Magnitude of Response. *Israel J. Sci.*, 7, 553.

Burnett, F. McF. (1969) *Self and Not-Self.* Melbourne: University Press.

Canales, L., Middlemas, R. O., Louro, J. M. & Smith, M. A. (1969) Immunological Observations in Chronic Mucocutaneous Candidiasis. *Lancet*, ii, 567.

Caspary, E. A. (1971) Lymphocyte–Antigen

Interaction in Electrophoretic Mobility Test for Cellular Sensitization. *Nature, Lond.*, (*New Biology*), **231**, 24.

CASPARY, E. A. & FIELD, E. J. (1965) An Encephalitogenic Proteion of Human Origin; Some Chemical and Biological Properties. *Ann. N.Y. Acad. Sci.*, **122**, 182.

CASPARY, E. A. & FIELD, E. J. (1971) Specific Lymphocyte Sensitization in Cancer: Is there a Common Antigen in Human Malignant Neoplasia? *Br. med. J.*, ii, 613.

CHU, E. H. Y., STJERNSWARD, J., CLIFFORD, P. & KLEIN, G. (1967) Reactivity of Human Lymphocytes against Autochthonous and Allogeneic Normal and Tumor Cells *in vitro*. *J. natn. Cancer Inst.*, **39**, 595.

COOPERBAND, S. R., BONDEVIK, H., SCHMID, K. & MANNICK, J. A. (1968) Transformation of Human Lymphocytes: Inhibition by Homologous Alpha Globulin. *Science, N.Y.*, **159**, 1243.

FIELD, E. J. (1972) Delayed Hypersensitivity Studies: Some Applications of Cell Electrophoresis. *J. R. Coll. Physicians Lond.*, in the press.

FIELD, E. J. & CASPARY, E. A. (1970) Lymphocyte Sensitization: an *in vitro* Test for Cancer. *Lancet*, ii, 1337.

FIELD, E. J., CASPARY, E. A. & BALL, E. J. (1963) Some Biological Properties of a Highly Active Encephalitogenic Factor Isolated from Human Brain. *Lancet*, ii, 11.

FIELD, E. J. & CASPARY, E. A. (1971) Demonstration of Sensitized Lymphocytes in Blood. *J. clin. Path.*, **24**, 179.

FIELD, E. J., CASPARY, E. A. & CARNEGIE, P. R. (1971) Lymphocyte Sensitization to Basic Protein of Brain in Malignant Neoplasia: Experiments with Serotonin and Related Compounds. *Nature, Lond.*, **233**, 284.

FRENSTER, J. H. & ROGOWAY, W. M. (1970) Immunotherapy of Human Neoplasms with Autologous Lymphocytes Activated *in vitro*. In *Proc. 5th Leukocyte Culture Conference*. Ed. J. E. Harris. New York: Academic Press. p. 359.

GATTI, R. A., GARRIOCH, D. B. & GOOD, R. A. (1970) Depressed PHA Responses in Patients with Non-lymphoid Malignancies. In *Proc. 5th Leukocyte Culture Conference*. Ed. J. E. Harris. New York: Academic Press. p. 339.

HEILMAN, D. H. & MACFARLAND, N. (1966) Inhibition of Tuberculin-induced Mitogenesis in Cultures of Lymphocytes from Tuberculous Donors. *Int. Archs Allergy appl. Immun.*, **30**, 58.

HUGHES, D. & CASPARY, E. A. (1970) Lymphocyte Transformation *in vitro* Measured by Tritiated Thymidine Uptake. *Int. Archs Allergy*, **37**, 506.

HUGHES, D. & PATY, D. W. (1972) Macrophage Migration Inhibition Test applied to Delayed Hypersensitivity in Man. I. A Mixed Lymphocyte Reaction between Human and Guinea Pig Lymphocytes Demonstrated using a Sensitive Micro-capillary Method. *Z. ImmunForsch. exp. Ther.*, **142**, 478.

HUGHES, D., CASPARY, E. A. & FIELD, E. J. (1968) Lymphocyte Transformation Induced by Encephalitogenic Factor in Multiple Sclerosis and Other Neurological Diseases. *Lancet*, ii, 1205.

KAMRIN, B. B. (1959) Successful Skin Homografts in Mature Non-littermate Rats Treated with Fractions Containing $\alpha$-globulins. *Proc. Soc. exp. Biol. Med.*, **100**, 58.

KNOWLES, M., HUGHES, D., CASPARY, E. A. & FIELD, E. J. (1968) Lymphocyte Transformation in MS—Inhibition of Unstimulated Thymidine Uptake by a Serum Factor. *Lancet*, ii, 1207.

LEHNER, T. (1970) Immunopathology of Oral Leukoplakia. *Br. J. Cancer*, **24**, 442.

LEVENE, G. M., TURK, J. L., WRIGHT, D. J. M. & GRIMBLE, A. G. S. (1969) Reduced Lymphocyte Transformation Due to a Plasma Factor in Patients with Active Syphilis. *Lancet*, ii, 246.

MACFARLIN, D. E. & OPPENHEIM, J. J. (1969) Impaired Lymphocyte Transformation in Ataxia-telangiectasia in Part Due to a Plasma Inhibitory Factor. *J. Immun.*, **103**, 1212.

MATHÉ, G., AMIEL, J. L., SCHWARZENBERG, L., SCHNEIDER, M., CATTAN, A., SCHLUMBERGER, J. R., HAYAT, M. & DE VASSAL, F. (1969) Active Immunotherapy for Acute Lymphoblastic Leukaemia. *Lancet*, i, 697.

MOWBRAY, J. F. (1963*a*) Effect of Large Doses of an $\alpha_2$-glycoprotein Fraction on the Survival of Rat Skin Homografts. *Transplantation*, **1**, 15.

MOWBRAY, J. F. (1963*b*) Ability of Large Doses of an $\alpha_2$ Plasma Protein Fraction to Inhibit Antibody Production. *Immunology*, **6**, 217.

MOWBRAY, J. F. & HARGRAVE, D. C. (1966) Further Studies on the Preparation of the Immunosuppressive alpha$_2$ Protein Fraction from Serum and its Assay in Mice. *Immunology*, **11**, 413.

PARONETTO, F. & POPPER, H. (1970) Lymphocyte Stimulation Induced by Halothane in Patients with Hepatitis following Exposure to Halothane. *New Engl. J. Med.*, **283**, 277.

RIGGIO, R. R., SCHWARTZ, G. H., BULL, F. G., STENZEL, K. H. & RUBIN, A. L. (1969) $\alpha_2$-globulins in Renal Graft Rejection. *Transplantation*, **8**, 689.

ROBINSON, E. & HURVITZ, D. (1966) *In vitro* Studies of Lymphocytes from Cancer Patients. *Israel J. med. Sci.*, **2**, 80.

SALK, M. (1967) Effect of Plasma from Patients with Carcinoma on *in vitro* Lymphocyte Transformation. *Cancer, N.Y.*, **20**, 2088.

SMITH, JUDITH K., CASPARY, E. A. & FIELD, E. J. (1972) Lymphocyte Reactivity to Antigen in Pregnancy. *Am. J. Obstet. Gynec.*, in the press.

TRUBOWITZ, S., MASEK, B. & DEL ROSARIO, A. (1966) Lymphocyte Response to Phytohaemagglutinin in Hodgkin's Disease, Lymphatic Leukemia and Lymphosarcoma. *Cancer, N.Y.*, **19**, 2019.